# SARS-CoV-2: Recent Variants and Clinical Efficacy of Antibody-Based Therapy

**DOI:** 10.3389/fcimb.2022.839170

**Published:** 2022-02-14

**Authors:** Desh Deepak Singh, Anshul Sharma, Hae-Jeung Lee, Dharmendra K. Yadav

**Affiliations:** ^1^ Amity Institute of Biotechnology, Amity University Rajasthan, Jaipur, India; ^2^ Department of Food and Nutrition, College of Bionanotechnology, Gachon University, Gyeonggi-do, South Korea; ^3^ Institute for Aging and Clinical Nutrition Research, Gachon University, Gyeonggi-do, South Korea; ^4^ Gachon Advanced Institute for Health Sciences and Technology, Gachon University, Incheon, South Korea; ^5^ Department of Pharmacy, Gachon Institute of Pharmaceutical Science, College of Pharmacy, Gachon University, Incheon, South Korea

**Keywords:** SARS-CoV-2, variant, antibody, treatment, efficacy, neutralization

## Abstract

Multiple variants of SARS-CoV-2 have emerged and are now prevalent at the global level. Currently designated variants of concern (VOCs) are B.1.1.7, B1.351, P.1, B.1.617.2 variants and B.1.1.529. Possible options for VOC are urgently required as they carry mutations in the virus spike protein that allow them to spread more easily and cause more serious illness. The primary targets for most therapeutic methods against SARS-CoV-2 are the S (Spike) protein and RBD (Receptor-Binding Domain), which alter the binding to ACE2 (Angiotensin-Converting Enzyme 2). The most popular of these strategies involves the use of drug development targeting the RBD and the NTD (N-terminal domain) of the spike protein and multiple epitopes of the S protein. Various types of mutations have been observed in the RBDs of B.1.1.7, B1.351, P. and B.1.620. The incidence of RBD mutations increases the binding affinity to the ACE2 receptor. The high binding affinity of RBD and ACE2 has provided a structural basis for future evaluation of antibodies and drug development. Here we discuss the variants of SARS-CoV-2 and recent updates on the clinical evaluation of antibody-based treatment options. Presently, most of the antibody-based treatments have been effective in patients with SARS-CoV-2. However, there are still significant challenges in verifying independence, and the need for further clinical evaluation.

**Graphical Abstract:**
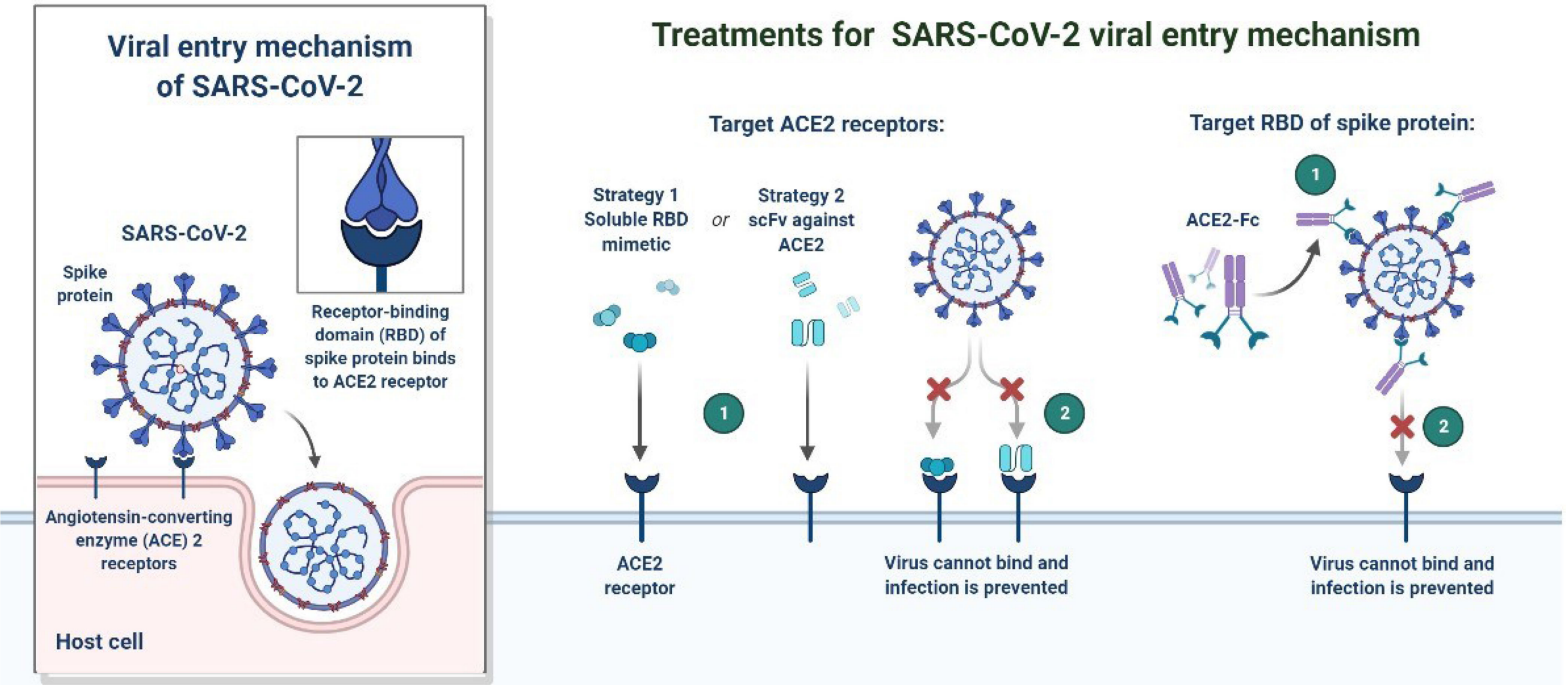


## Introduction

**Figure 1:**
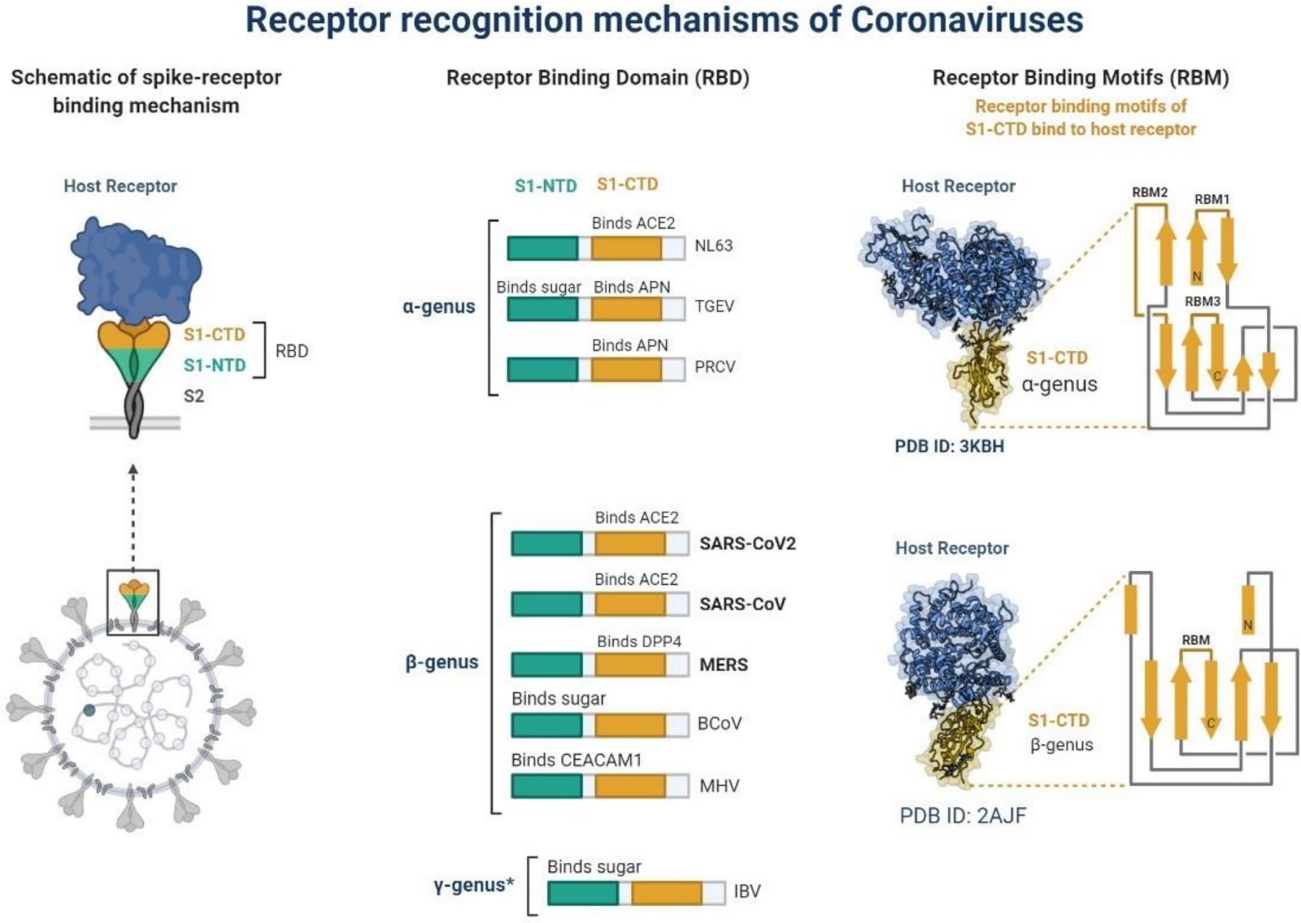
Schematic representation of receptor recognition and binding, spike receptor binding mechanism, receptor binding domain and receptor binding motifs. * γ-genus is belong to Avian infectious bronchitis virus (IBV).

In December 2019, a non-specific case of respiratory disorder was reported in Wuhan, Hubei Province, Republic of China, and it was transmitted from human to human (Chen et al., 2020). SARS-CoV-2, a coronavirus, is found in more than 200 nations and territories around the world. Coronaviruses are divided into four groups: Alpha (B.1.1.7), Beta (B1.351), Gamma (P.1), Delta (D.1) and Omicron (B.1.1.529). Human coronaviruses are Alpha and Beta coronaviruses (Singh et al., 2020a). Bats are hosts to the largest number of viral genotypes of coronaviruses. Coronaviruses and their characteristics are shown in **Table 1** (Singh et al., 2020b). SARS-CoV-2 has genetic markers that have been linked to a potentially increased risk (Singh et al., 2021). New variants may elude medical treatments (Haimei, 2020). Researchers from the field at a global level were notified of the emergence of a SARS-CoV-2 variant (Kar et al., 2021). More than half of the total genomic sequencing of SARS-CoV-2 was carried out in the UK. Researchers from the field have identified eight global clades and classified them as S, O, L, V, G, GH, GR, and major lineages such as A, B, B.1, B.1.1, B.1.177, and B.1.1.7 have been identified (Koyama et al., 2020; Nayak et al., 2021). The Omicron variant, known as lineage B.1.1.529, was proclaimed a variant of concern by the World Health Organization on November 26, 2021 (Callaway, 2021). There are over 30 mutations in the variant, some of which are worrisome. The number of cases in line B.1.1.529 is increasing in all regions of South Africa. First discovered in South Africa, this new strain is now spread to more than 10 countries, including Canada, the United Kingdom, the Netherlands, Denmark and Australia. Concerns are growing around the world that the new strain will be more resistant to vaccine protection, prompting concerns that the pandemic and associated lockdown restrictions will last considerably longer than planned (Callaway, 2021). Research on Omicron has begun around the globe, but it is not yet clear if this new COVID variant is more transmissible than other previous variants such as Alpha, Kappa, Delta, etc. (Callaway, 2021). Mutations found in other VOCs include the N501Y mutation, which improves the binding of peplomer proteins to cell receptors, and the D614G mutation, which is thought to increase viral replication, both of which can increase viral infectivity. There is a sex. Others include the K417N and T478K mutations. These help the virus evade neutralizing antibodies produced by vaccination or previous infections. Researchers have discovered B.1.1.529 with 43 peplomer mutations in Rome (Callaway, 2021). The SARS-CoV-2 protein recognizes host cells and is the primary target of the body’s immune response. In November, cases increased rapidly in many countries, especially schools and adolescents. Variants have spike mutations that allow detection by genotyping tests that provide much faster results than genomic sequencing. The new variant of coronavirus reportedly has more than 30 mutations in the spike protein region and therefore has the potential to develop immune escape mechanisms. Most vaccines form antibodies against the spike protein, and so many mutations in the spike protein region may lead to a decreased efficacy of therapeutic options. The effectiveness of SARS-CoV-2 therapeutic developments is affected by the new emergent variants at the global level. Antibodies against the surface of the SARS-CoV-2 are commonly used to neutralize infection (Wang et al., 2020; Diamond et al., 2021). Most of the drugs are targeted towards the receptor binding domain (RBD) of the spike protein, and multiple epitopes of the S protein, as shown in **Figure 1**.

**Table 1:** List of important pathogenic coronaviruses their host organisms, genera name, and associated clinical manifestations.

S. No.	Name	Host organism	Genera name	Clinical manifestations
1	Feline infectious peritonitis virus	Cat	Alpha	Vasculitis, fever, serositis, with or without effusions
2	Camel alphacoronavirus isolate camel/Riyadh	Camel	Alpha	Asymptomatic
3	Canine CoV/TU336/F/2008	Dog	Alpha	Diarrhea and mild clinical signs
4	SeACoV-CH/GD-01	Pig	Alpha	Acute and severe diarrhea and vomiting
5	TGEV/PUR46-MAD	Pig	Alpha	Diarrhea
6	PRCV/ISU-1	Pig	Alpha	Mild respiratory tract infections (RTIs)
7	PEDV/ZJU-G1-2013	Pig	Alpha	Severe watery diarrhea
8	Human CoV-NL63	Human	Alpha	Mild RTIs
9	Human CoV-229E	Human	Alpha	Mild RTIs
10	MHV-A59	Mouse	Beta	Severe lung injuries and acute pneumonia
11	Equine CoV/Obihiro12-1	Horse	Beta	Leucopenia, fever, and anorexia
12	Bovine CoV/ENT	Cow	Beta	Diarrhea
13	MERS-CoV	Human	Beta	Severe acute respiratory syndrome
14	SARS-CoV	Human	Beta	Severe acute respiratory syndrome
15	Human CoV-OC43	Human	Beta	Mild RTIs
16	Human CoV-HKU1	Human	Beta	Pneumonia
17	IBV	Chicken	Gamma	Severe respiratory disease
18	Beluga Whale CoV/SW1	Whale	Gamma	Terminal acute liver failure and pulmonary disease
19	Sparrow coronavirus HKU17	Sparrow	Delta	Respiratory disease
20	Bulbul coronavirus HKU11	Bulbul	Delta	Respiratory disease

As the protein SARS-CoV-2 and its RBD have been shown *in vitro* in cell culture, neutralization of the mAb against them effectively inhibits the binding of the virus to the host receptor, human angiotensin converting enzyme (hACE2), and thus is a major target of the mAb. Blocks viruses from invading cells (Yang et al., 2021). Some antibodies bound outside the RBD may also neutralize the virus *in vitro* using an undefined mechanism. Some of the neutralizing antibodies passively protect SARS-CoV-2 infected animal models with high efficacy (Sette and Crotty, 2021). Longitudinal studies evaluating the onset and duration of viral shedding and antibody response are needed in asymptomatic, mild, or severe patients. Here we discuss the emergence of variants of SARS-CoV-2 and the clinical evaluation of antibody-based treatment options. Presently, most of the antibody-based treatments have been effective in patients with SARS-CoV-2. However, there are still significant challenges in verifying independence, and a need for further clinical evaluation.

## Antibody-Based Treatment Options

The appearance of novel SARS-CoV-2 variants has been observed all over the world, hampering the drug development process (**Tables 2**, **3**). New variants of current therapeutic options are required to maintain clinical efficacy (Sette and Crotty, 2021). More clinical investigations are required for FDA approval against emerging variants. Bamlanivimab and etesevimab, will expected stagger in efforts to improvement full FDA approval given the antiviral resistance observed against B.1.351, P.1. and B.1.526 (Doggrell, 2021). Optimization are required for its monoclonal antibody (mAb) to prove effective against the UK B.1.1.7 variant. Bamlanivimab have been observed less effective against most of the variants, but improved efficacy was observed in combination with etesevimab (Focosi et al., 2021). The FDA has cancelled the EUA for bamlanivimab as monotherapy. Combo of casirivimab/imdevimab has been observed more effective against new variants of SARS-CoV-2. Phase-III clinical trial data of casirivimab and imdevimab has been observed effective against new variants (Taylor et al., 2021).

**Table 2:** Relative risk level for variants of concern (VOC).

Identification	WHO level	Alpha	Alpha	Beta	Delta	Gamma
	**Phylogenetic Assignment of Named Global Outbreak (PANGO) Lineages**	B.1.1.7	Alpha with E484K	B.1.351	B.1.617.2	P.1
	**Public Health England (PHE)**	VOC−20DEC−01	VOC−21Feb−02	VOC−20DEC−02	VOC−21APR−02	VOC−21JAN−02
	**Nextstrain clade**	20I (V1)	20I (V1)	20H (V2)	21A	20J (V3)
**Emergence**	**First outbreak**	United Kingdom	United Kingdom	South Africa	India	Brazil
	**Earliest sample**	20 Sep 2020	26 Jan 2021	May 2020	Oct 2020	Nov 2020
	**Designated VOC**	18 Dec 2020	5 Feb 2021	14 Jan 2021	6 May 2021	15 Jan 2021
**Changes relative to previously circulating variants at the time and place of emergence**	**Notable mutation**	69–70del, N501Y, P681H	E484K, 69–70del, N501Y, P681H	K417N, E484K, N501Y	L452R, T478K, P681R	K417T, E484K, N501Y
	**Transmissibility**	+29% (24–33%)	+29% (24–33%)	+25% (20–30%)	+97% (76–117%)	+38% (29–48%)
	**Hospitalization**	+52% (47–57%)	+52% (47–57%)	Under investigation	+85% (39–147%)	Possibly increased
	**Mortality**	+59% (44–74%) CFR 0.06% for <50 age group, 4.8% for >50 age group	+59% (44–74%) CFR 0.06% for <50 age group, 4.8% for >50 age group	Possibly increased	+137% (50–230%) CFR 0.04% for <50 age group unvaccinated, 6.5% for >50 age group unvaccinated	+50% (50% CrI, 20–90%)
**Neutralizing antibody activity**	**From natural infection**	Minimal reduction	Considerably reduced	Reduced, T cell response elicited by D614G virus remains effective	Reinfections happened, with smaller occurrence rate than vaccinated infections	Efficacy reduction for non-severe disease
	**Vaccination**	Minimal reduction	Considerably reduced	Efficacy: reduced against symptomatic disease, retained against severe disease	Efficacy reduction for non-severe disease	Retained by many

**Table 3:** List of variants for further monitoring.

Pango lineage	GISAID clade	Date of designation	Comments
R.1	GR	07-04-2021	It has found in more than 30 countries, E484K and W152L mutation have been observed, it may cause immune escape.
B.1.466.2	GH	28-04-2021	First sampled in Indonesia, in Nov 2021.
B.1.1.318	GR	02-06-2021	Detected in the UK, it was named Fin-796H after found in Finland with E484K and D796H mutations originate from Nigeria.
B.1.1.519	GR	02-06-2021	Variants Under Monitoring (VUM) in Nov 2021.
C.36.3	GR	16-06-2021	VUM in Nov 2021.
B.1.214.2	G	30-06-2021	VUM in Nov 2021.
B.1.427 B.1.429	GH/452R.V1	06-07-2021	VUM in Nov 2021. Epsilon, first sample was observed in the United States.
B.1.1.523	GR	14-07-2021	VUM in Nov 2021.multiple countries
B.1.619	G	14-07-2021	VUM in Nov 2021.multiple countries
B.1.620	G	14-07-2021	Detected in Lithuania, Central Africa, North America, France and Belgium, the lineage contains an E484K, P681H, S477N and D614G mutation
C.1.2	GR	01-09-2021	It was detected in England and China, Portugal, Switzerland, Democratic Republic of the Congo (DRC), Mauritius, and New Zealand with multiple substitutions C136F, R190S, D215G, Y449H, N484K, N501Y, H655Y, N679K and T859N and deletions (Y144del, L242-A243del) in the spike protein.
B.1.617.1	G/452R.V3	20-09-2021	Kappa
B.1.562	GH/253G.V1	20-09-2021	Iota
B.1.525	G/484K.V3	20-09-2021	Eta
B.1.630	GH	12-10-2021	Identified in March 2021, Dominican Republic.
B.1.1.529	GR/484A, 200	24-11-2021	Named Omicron by the WHO, identified in November 2021 in more than 15 countries.

## Sotrovimab

Sotrovimab (VIR-7831), an antibody drug, is based on the entry of coronavirus into the body. Data from phase III clinical trials revealed that this medicine lowers the rate of hospitalization and death (Gupta et al., 2021). In a recent study published in The New England Journal of Medicine, researchers theorized that a monoclonal antibody that neutralizes all SARS-CoV-2 would target a highly conserved epitope that would remain effective as SARS-CoV-2 mutates (Aschenbrenner, 2021). In the phase III, multicenter, double-blind, placebo-controlled study, SARS-CoV-2 Monoclonal Antibody Efficacy Trial–Intent to Care Early (COMET-ICE), Researchers evaluated the impact of a single intravenous infusion of sotrovimab 500 mg on mild-to-moderate SARS-CoV-2 in high-risk, non-hospitalized patients (Cheng et al., 2021). The risk of severe SARS-CoV-2 is higher in patients over 55 years old or in those who have diabetes, obesity, chronic kidney disease, chronic obstructive pulmonary disease, congestive heart failure, or moderate-to-severe asthma. One-time infusions of 500 mg of sotrovimab or placebo saline were given randomly to the patients (1:1). Primary outcomes were the percentage of patients who died or spent more than 24 hours in the hospital. A 72-day follow-up was averaged for the sotrovimab and placebo groups in the intention-to-treat population. Overall, 1% (3/291) of patients in the sotrovimab group and 7% (21/292) of patients in the placebo group had disease progression requiring hospitalization or death. In high-risk adults with symptomatic SARS-CoV-2, a single 500 mg dose of sotrovimab was found to minimize the probability of hospitalization or mortality by 85% (Aschenbrenner, 2021; Cheng et al., 2021; Gupta et al., 2021). COMET-ICE, which compared monoclonal antibodies to SARS-CoV-2 and the subsequent variants, was apparent as evidence that sotrovimab neutralized SARS-CoV-2 and its variants. Sotrovimab has also shown efficacity against variant lineages B.1.1.7, B.1.351, P.1, B.1.617, B.1.427/B.1.429 and B.1.526. Preclinical data suggest it could both block viral entry into healthy cells and clear infected cells by binding to an epitope on SARS-CoV-2 that’s participated with SARS-CoV-1 (Cheng et al., 2021; Gupta et al., 2021).

## Lenzilumab

Lenzilumab is an engineered anti-human granulocyte-macrophage colony-stimulating factor (GM-CSF) monoclonal antibody designed to prevent and treat cytokine release syndrome preceding lung dysfunction and acute respiratory distress syndrome in serious SARS-CoV-2 infection cases (Bonaventura et al., 2020; Temesgen et al., 2021b). Lenzilumab aced the Phase III LIVE-AIR trial (NCT04351152), a 54 relative enhancement in the liability of survival without ventilation (SWOV) vs. placebo (Temesgen et al., 2021a). SWOV liability bettered by 92 in actors who entered both corticosteroids and Gilead Lores remdesivir (Veklury), and triple in cases under 85 times of age with a C-reactive protein position of< 150 mg/L. In the NIAID- patronized, placebo- controlled Phase II ACTIV-5 Big Effect Trial (NCT04583969), lenzilumab is being studied alone and in combination with Veklury to help and treat cytokine storms. Lenzilumab is also being researched for a variety of other indications. In May, Lenzilumab Humanigen submitted an application to the FDA for an emergency use authorization (EUA) for lenzilumab to treat SARS-CoV-2 patients hospitalized. Lenzilumab has been proven to be effective against the B.1.1.7, P.1, B.1.617, B.1.427/B.1.429, and B.1.526 variant lineages (Bonaventura et al., 2020;Temesgen et al., 2021b).

## Bamlanivimab

Bamlanivimab (LY-CoV555) is a recombinant human IgG1 mAb, that prevents viral attachment and penetration into human cells while also neutralizing the virus (Kuritzkes, 2021). The Journal of the American Medical Association released the results of a Phase 3 study of bamlanivimab among residents and staff in long-term care facilities (NCT04497987) (Dougan et al., 2021). The emergency use of LY-CoV555 700 mg in combination with etesevimab (LY-CoV016) 1400 mg has been expanded by the FDA to include post-exposure prophylaxis (PEP) to prevent SARS-CoV-2 infection or symptomatic SARS-CoV-2 infection (**Table 4**). These antibodies, which have been demonstrated to be effective against the extremely contagious Delta variant, can now be used to protect some of the most vulnerable people who are exposed to the virus with this expanded authorization (Dougan et al., 2021; Nathan et al., 2021). Bamlanivimab and etesevimab jointly retain neutralizing activity against the Alpha and Delta forms, according to pseudovirus and authentic virus studies. Because both the P.1 and B.1.351 variants exhibit reduced sensitivity to bamlanivimab and etesevimab, the distribution of bamlanivimab with etesevimab has been halted in the United States. However, in areas with low prevalence of these and other variants that have lowered susceptibility to bamlanivimab and etesevimab, the distribution of the agents has been reinstated in states (Dougan et al., 2021; Nathan et al., 2021).

**Table 4:** Recent updates on clinical data of anti-SARS-CoV-2 selected monoclonal antibodies.

Double-blind, randomized controlled trial in SARS-CoV-2 patients with mild- to -moderate	Phase	Dose concentration	Inclusion criteria	Interventions compared to placebo	Participant characteristics	Interpretation (compared to placebo)	Primary endpoint	Primary outcomes (SARS-CoV-2-related hospitalizations over days)
Bamlanivimab (BAM)	Double-Blind, Phase 3	700 mg + Etesevimab + 1,400 mg in Nonhospitalized	Aged ≥12 years	BAM 700 mg + ETE 1,400 mg (*n* = 511) Within 3 days of a positive SARS-CoV-2, Placebo (*n* = 258).	Median age 56 years; 30% ≥65, 76% mild and 24% had moderate SARS-CoV-2 patient.	5% absolute reduction and 87% relative reduction in SARS-CoV-2-related hospitalizations.	defined as ≥24 hours of acute care.	Day 29: 0 in BAM plus ETE arm vs. 4 (1.6%) in placebo arm; *P* = 0.01.
			At high risk for severe SARS-CoV-2 patient.					
Bamlanivimab with Etesevimab	Phase 3	Bamlanivimab 2,800 mg Plus Etesevimab (ETE) 2,800 mg in Nonhospitalized patients	Aged ≥12 years	In 3 days of a positive SARS-CoV-2 patient, BAM 2,800 mg with ETE 2,800 mg (*n* = 518); Placebo (*n* = 517).	Mean age 53.8 years; 31% ≥65 years; 52% female; 48% male	Placebo with 4.8% absolute reduction and 70% relative in hospitalized patients.	Proportion of patients with SARS-CoV-2-related hospitalization	Day 7: 9.8% in BAM plus ETE arm vs. 29.5% in placebo arm (*P* < 0.001)
			At high risk for severe SARS-CoV-2 or hospitalization					
Casirivimab (CAS) Plus Imdevimab (IMD) in Nonhospitalized	Phase 3	Aged ≥18 years with SARS-CoV-2 positive; Symptom onset within 7 days of randomization; analysis only: ≥1 risk factor for severe SARS-CoV-2.	Single IV (intravenous)infusion of CAS 600 mg with IMD 600 mg (*n* = 736) or placebo (*n* = 748); CAS 1,200 mg plus IMD 1,200 mg (*n* = 1,355) or placebo (*n* = 1,341).	CAS 600 mg plus IMD 600 mg (*n* = 736) or placebo (*n* = 748), CAS 1,200 mg plus IMD 1,200 mg (*n* = 1,355) or placebo (*n* = 1,341).	Median age 50 years; 35% Hispanic/Latinx; 5% Black/African American.	CAS 600 mg with IMD 600 mg was associated with 2.2% absolute reduction and 70% relative risk reduction in SARS-CoV-2 Patients.	Proportion of patients with SARS-CoV-2-related hospitalization through Day 29.	Day 29,7 (1.0%) in CAS 600 mg with IMD 600 mg arm vs. 24 (3.2%) in placebo arm (*P* = 0.002). 18 (1.3%) in CAS 1,200 mg plus IMD 1,200 mg arm vs. 62 (4.6%) in placebo arm (*P* < 0.001).
Sotrovimab (SOT) in Non-hospitalized patients with mild -to- moderate SARS-CoV-2	Phase -III	SOT 500 mg, Placebo (*n* = 292)	Aged ≥18 years with ≥1 comorbidity, aged ≥55 years, Symptom onset ≤5 days Laboratory-confirmed SARS-CoV-2.	SOT 500 mg IV (*n* = 291)	Median age 53 years; 22% ≥65 years	Receipt of SOT was associated with 6% absolute reduction and 85% relative risk reduction.	Proportion of patients with all-cause hospitalization or death by Day 29	Day 29: 3 (1%) in SOT arm vs. 21 (7%) in placebo arm (*P* = 0.002).
				Placebo (*n* = 292)	63% Hispanic/Latinx; 7% Black/African American			

## AZD7442

AstraZeneca has developed two antibody cocktails known as AZD7442, which have been shown to have potent responses against SARS-CoV-2 (Mahase, 2021). In a clinical trial including 5000 volunteers, AZD7442 was reported to be 77% effective in a patient with SARS-CoV-2. AstraZeneca reported the results in August 2021 (Dong et al., 2021). That continuity would make it especially useful to immunocompromised cases who do not get important protection from vaccines (Dong et al., 2021). The federal government has reached an agreement with the company to order up to 700,000 doses of the treatment this year, but it will first need to be authorized (Dong et al., 2021). AZD7442 has been shown to be effective against the variant lineages B.1.1.7, P.1, B.1.617, B.1.427/B.1.429, and B.1.526 (Dong et al., 2021).

## BRII-196/BRII-198

BRII-196/BRII-198 is a SARS-CoV-2 negativing monoclonal antibody combination remedy (Yang et al., 2020). Preliminary *in vitro* evidence suggests continued antiviral activity against commonly circulating variants from the U.K. and South Africa (Yang et al., 2020). A phase 1 study completed dosing and follow-up by providing safety profiles and human pharmacokinetic profiles for two separate antibodies (Baral et al., 2021). Combination therapy consisting of BRII196 and BRII198 was originally investigated in the April 2021 NIAID ACTIV3 study (NCT04501978) in inpatients. However, it did not meet the pre-determined performance criteria required for Phase 3 entry. As part of an ongoing NIH ACTIV2 trial (NCT04518410), a mixture of BRII196 and BRII198 antibodies is in phase 3 clinical trials (Baral et al., 2021).

## CERC-002

CERC002 is a fully human monoclonal antibody against LIGHT or TNFSF14 (a member of the tumor necrosis factor superfamily 14) (Haljasmägi et al., 2020). He is currently being tested for SARS-CoV-2 ARDS due to Crohn’s disease and cytokine storm. This study will evaluate the efficacy and safety of CERC002 in patients with severe SARS-CoV-2 for 28 days as a single dose in addition to standard treatment (Perlin et al., 2020). CERC002 increased survival by day 28 and the number of people without respiratory failure in hospitalized patients with mild to moderate SARS-CoV-2-associated pneumonia (ARDS) compared to placebo (83.9% versus 64.5%, *P* = 0.044). Efficacy was highest in the predefined patient subgroup 60 years and older (76.5% versus 47.1%, *P* = 0.042), which is the population most vulnerable to serious complications and death from SARS-CoV-2 infection (Rodriguez-Perez et al., 2021). There was an approximately 50% reduction in mortality with CERC002 compared to placebo on both the first 28 days and 60 days (7.7% vs. 14.3% at 28 days and 10.8% vs. 22.5% at 60 days) (Rodriguez-Perez et al., 2021). In the final efficacy data for the phase 2 study (NCT04412057), Cerecor showed that more COVID19 patients with acute respiratory distress who received a single dose of CERC002 instead of placebo were alive and not experiencing dyspnea during the 28-day study period. The efficacy was highest in patients over the age of 60 who frequently suffered from other inflammatory diseases.

## SAB-185

SAB185 is a therapeutic candidate for neutralizing polyclonal antibodies to treat non-hospitalized mild-to-moderate SARS-CoV-2 patients (Winkler et al., 2021). The candidate is being evaluated in the ACTIV2 trial conducted by NIAID, which is part of the NIH in collaboration with the AIDS Clinical Trial Group. SAB185 is a fully human polyclonal candidate antibody designed to confer passive immunity. The first patient in the NIAID-sponsored Phase II/III ACTIV2 study (NCT04518410) received a dose of SAB185 in April after a previous trial demonstrated the safety of an antibody with a half-life of 25-28 days. SAB185, the second drug to enter Phase 3 and the first candidate for polyclonal antibody therapy in ACTIV2, is evaluating several research drugs to treat early symptoms of SARS-CoV-2 in non-hospitalized individuals (Liu et al., 2021). SAB185 was transferred to Phase II as part of the Phase III ACTIV2 trial after meeting all required termination criteria. SAB185 effectively neutralizes viruses containing SARS-CoV-2 spikes with S477N, E484K, and N501Y mutations. This virus has been associated with the outbreak and outbreak of SARS-CoV-2 in several countries, leading to antibody resistance (Liu et al., 2021). WHO has identified several VOCs with mutations in the spike protein SAB185 was tested in BSL2 medium using a lentiviral pseudo virus experiment containing a stable 293T cell line expressing human ACE2 and TMPRSS2. Data collected from 221 patients in study SG016 Phase II (NCT04385095) showed that 33 patients with severe or severe dyspnoea and received SNG001 were 3.41 times more likely to recover than patients who received placebo. *In vitro* data show that SNG001 exhibits antiviral activity against two strains of COVID19, B.1.1.7 and B.1.351 (Saeed et al., 2020). The results show that SAB185 retained neutralizing ability against several strains of SARS-CoV-2-like virus, including delta, kappa and lambda variants, which is displacing other VOCs in many countries and regions around the world (Liu et al., 2021).

## Casirivimab/Imdevimab

In outpatients with mild to moderate SARS-CoV-2, a placebo-controlled randomized trial looked at different dosages of casirivimab plus imdevimab (Razonable et al., 2021). FDA simplified the EUA for casirivimab plus imdevimab, reducing the approved dose for single intravenous infusion from casirivimab 1200 mg plus imdevimab 1200 mg to casirivimab 600 mg plus imdevimab 600 mg (NCT04425629) (Deeks, 2021; Razonable et al., 2021). Participants included were 18 years of age or older, tested positive for SARS-CoV-2, and had at least one risk factor for developing severe SARS-CoV-2. Results showed a 2.2% overall reduction and a 70% reduction in hospitalizations or deaths when taking casirivimab 600 mg plus imdevimab 600 mg. These results are similar to those observed with an intravenous infusion of casirivirab 1200 mg plus imdevimab 1200 mg, which resulted in an absolute 3.3° reduction in hospitalizations or deaths and a 71% relative decrease (NCT04519437), also found to be active against delta variant (O’Brien et al., 2021).

## Regdanvimab

Regdanvimab (CTP59) blocks the RBD interaction region of ACE2 in one direction. Therefore, CTP59 has the potential to be a promising treatment candidate for COVID19 (Kim et al., 2021). In September 2021, the Korean Ministry of Food and Drugs (MFDS) treated patients over the age of 50 with mild COVID19 and approved Regdanvimab in adults with at least one underlying disease and moderate disease symptoms. This approval is based on the first part of a global Phase 2/3 study showing a 54% reduction in progression to severe COVID 19 in patients with mild to moderate symptoms and a 68% reduction in patients over 50 years of age. In October 2021, the European Medicines Agency (EMA) began considering a marketing authorization application for this mAb for the treatment of adults with COVID19 who do not require additional oxygen therapy and are at high risk of developing severe COVID19. Did. The dose of Regdanvimab is a single intravenous infusion of 40 mg/kg (Syed, 2021). A double-blind, placebo-controlled, randomized, phase II study, BLAZE4 (NCT04634409), found the efficacy of other mAbs, including bumlanivimab (700 mg) and sotrovimab (500 mg), for the treatment of symptoms. We are evaluating safety. Low-risk, non-hospitalized COVID 19 patients. Preliminary results showed that bablanivimab/sotrovimab (700/500 mg) showed 70% (p <0> 5.27; day 7 cycle threshold <27.5 vs. placebo) (Syed, 2021). A recent study compared and evaluated all published studies investigating SARS-CoV-2 neutralized mAbs (single or combined vs. active comparator, placebo, or no intervention) for the treatment of patients with COVID19 and “to the evidence. I evaluated “trust”. About preventive use). The authors conclude that the available evidence is insufficient to draw meaningful conclusions about treatment with SARS-CoV-2-neutralized mAbs (Kim et al., 2021; Syed, 2021)

## Interferons

Interferons are produced by our cells naturally against viral infection. Interferons have strong effects on the immune system, stimulating it to attack invaders while also inhibiting it to avoid damaging the body’s own tissues (Felgenhauer et al., 2020). Injecting synthetic interferons is now a standard treatment for several immune disorders. Interferon’s approach to storming our bodies, enthused researchers to see whether an improvement in interferon might help in the early-stage infection of patients with SARS-CoV-2 (Della-Torre et al., 2020). Preliminary investigations in cells and mice have yielded reassuring results that have led to clinical trials (Murugan et al., 2021). On October 20, 2021, Synairgen proclaimed that the drug was moving forward into a Phase III clinical trial in mild- to -moderate SARS-CoV-2 patients. Sarilumab and tocilizumab are two classes of FDA-approved IL-6 inhibitors (**Table 5**).

**Table 5:** Recent updates on clinical evaluation of selected interleukin-6 inhibitors.

Open-Label RCT in hospitalized patients with SARS-CoV-2	Key inclusion criteria	Participant characteristics (PCR-confirmed SARS-CoV-2 infection)	Key limitations	Interventions	Primary outcomes	Key secondary endpoints
Tocilizumab	Oxygen saturation (SpO2) <92% on room air or receipt of supplemental oxygen	Mean age 63.6 years; 67% male; 76% White, 41% on HFNC or non-invasive ventilation,14% on IMV,82% on corticosteroids.	Arbitrary enrollment cut off at CRP ≥75 mg/L	800 mg tocilizumab) and probable second dose (*n* = 2,022), Usual care (*n* = 2,094).	Day 28 mortality was lower in tocilizumab arm than in usual care arm (31% vs. 35%; rate ratio 0.85; 95% CI, 0.76–0.94; *P* = 0.003)	Among those not on IMV at enrollment, receipt of IMV (invasive mechanical ventilation) or death.
	C-reactive protein (CRP)≥75 mg/L		Difficult to define exact subset of patients in RECOVERY cohort who were subsequently selected for secondary randomization/tocilizumab trial			
Tocilizumab and Sarilumab	Receipt of IMV, noninvasive ventilation, or cardiovascular support.	Mean age 60 years; Median time from ICU admission until enrollment was 14 hours	Enrollment in tocilizumab and sarilumab arms was partially nonconcurrent with SOC (Standard of care) arm.	Tocilizumab 8 mg/kg and possible second dose, plus SOC (*n* = 952)	1.46 (95% CrI, 1.13–1.87).	66% in tocilizumab arm and 63% in SOC arm (aOR 1.42; 95% CrI, 1.05–1.93).
Sarilumab	Aged ≥18 years; SARS-CoV-2 pneumonia.	Median age 59 years; 63% male; 77% White; 36% Hispanic/Latinx; 39% on HFNC, IMV, or non-invasive mechanical ventilation.	Only 20% of patients received corticosteroids.	There was no benefit of sarilumab in hospitalized adults with SARS-CoV-2 in time to clinical improvement.	No difference in median time to clinical Improvement among the sarilumab arms.	(92% in placebo arm vs. 90% in sarilumab 200 mg arm vs. 92% in sarilumab 400 mg arm).

## Sarilumab

Sarilumab is a monoclonal antibody that has been evaluated for off-label usage in the treatment of SARS-CoV-2. It binds to both membrane-bound and soluble IL-6 receptors with significant affinity (Gremese et al., 2020). Sarilumab 400 mg is reconstituted in 100 cc of 0.9% NaCl and administered as an hour long IV infusion. The SQ formulation was utilized to produce the IV infusion in the randomized, embedded, multifactorial adaptive platform trial for community-acquired pneumonia (REMAP-CAP) trial. In a revised report of the REMAP-CAP trial, sarilumab and tocilizumab were equally effective in improving survival and reducing time to organ supply. Patients receiving dexamethasone and sarilumab had lower mortality than patients in the control group who received dexamethasone plus placebo, had shorter time to discharge from the ICU, and had more days without organ support (https://www.covid19treatmentguidelines.nih.gov/). The combination of sarilumab and dexamethasone (*n* = 483) is 99% and 98% likely to be inferior to tocilizumab (*n* = 943) with dexamethasone in terms of days without organ support and days of death, respectively. REMAPCAP studies have shown that tocilizumab and sarilumab show similar efficacy in treating inpatients with COVID 19, but the panel recommends the use of sarilumab only if tocilizumab is not available or applicable. A single 400 mg dose of sarilumab for injection of SQ was reconstituted with normal saline (50 or 100 ml) and intravenously over 1 hour in the REMAPCAP study (https://www.covid19treatmentguidelines.nih.gov/). It was administered as an internal infusion. Recommendations for COVID19 treatment for the IL6 inhibitors sarilumab and tocilizumab in hospitalized patients requiring oxygenation, high flow oxygen, non-invasive ventilation or invasive ventilation (https://www.covid19treatmentguidelines.nih.gov/).

## Tocilizumab

**Figure 2:**
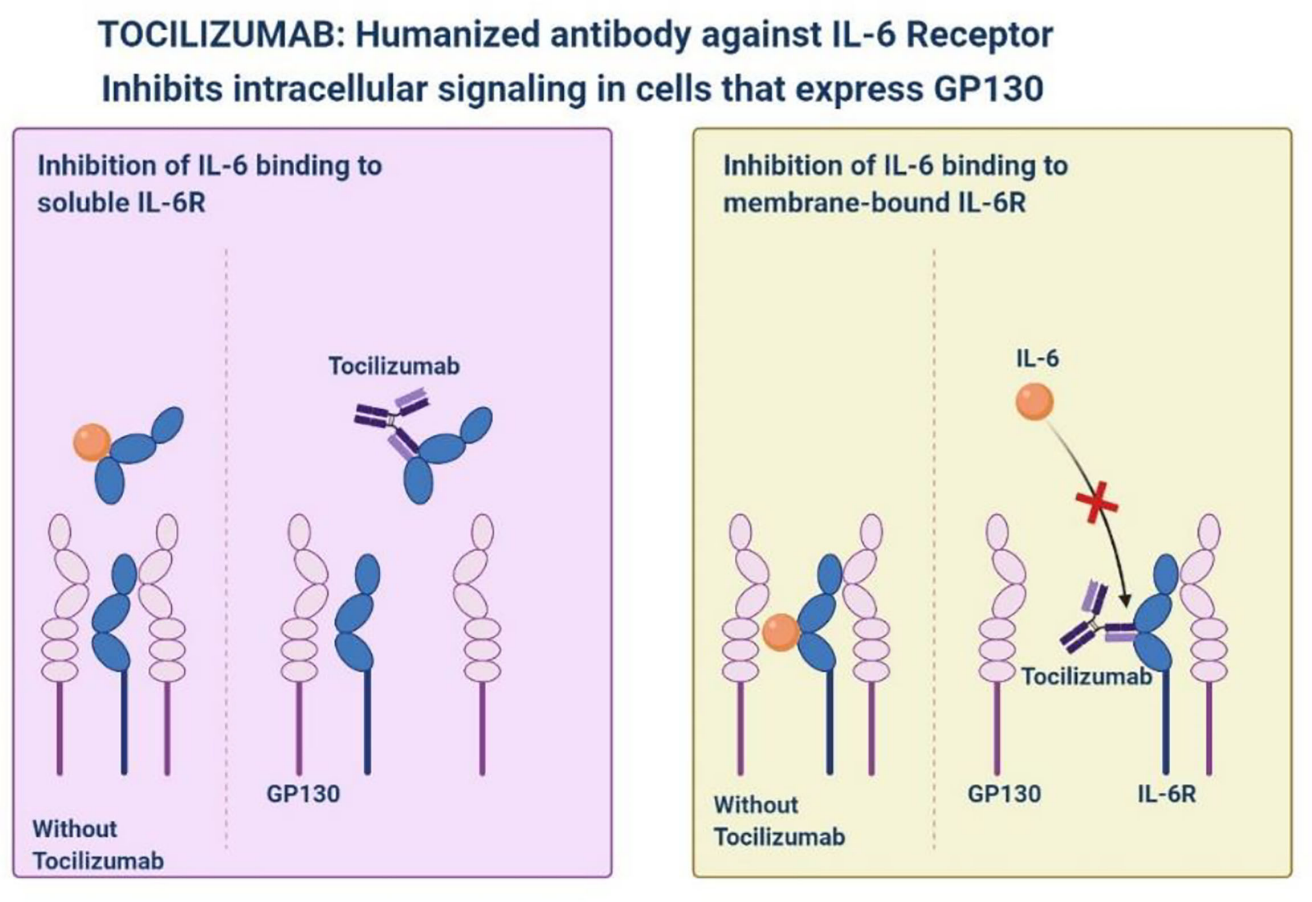
Inhibition of intracellular signaling by Tocilizumab Humanized Antibody against IL-6.

Tocilizumab is a monoclonal antibody against interleukin-6 receptor-alpha that is used for inflammatory diseases, improved consequences have been observed in patients with severe SARS-CoV-2 pneumonia (**Figure 2**) (Samaee et al., 2020; Stone et al., 2020). Tocilizumab showed a slower progression of the disease, as well as a sharp decrease in temperature and mechanical ventilation. In the STOPCOVID study, tofacitinib was associated with a lower risk of respiratory failure and death (hazard ratio 0.63, 95% CI 0.41–0.97). Within 28 days, 5.5% of patients in the placebo group (*n* = 145) and 2.8% of patients in the tofacitinib group (*n* = 144) (hazard rate 0.49, 95% CI, 0.15-1.63) had all-cause mortality. About 80% of participants in each group also received corticosteroids. Serious adverse events occurred in 14.2% of participants in the tofacitinib group and 12.0% of participants in the placebo group (Hermine et al., 2021). STOPCOVID study found that tofacitinib plus steroids improved outcomes in hospitalized SARS-CoV-2 patients.

## A Monoclonal Antibody Approved Against SARS-CoV-2 by Emergency Use Authorizations (EUAs)

EUA of mAbs against SARS-CoV-2 were due to the context declared emergency without available alternatives (Aschenbrenner, 2021). EUA is a mechanism used by the FDA to facilitate making products available quickly during a public health emergency; this differs from FDA approval, which is an independent, scientifically reviewed approval for medical products, drugs, and vaccines, based on substantial clinical data and evidence (Bonaventura et al., 2020; Temesgen et al., 2021b). The use of SARS-CoV-2 neutralizing antibodies has not been authorized by the FDA-EUAs for patient hospitalized for SARS-CoV-2 or for those requiring oxygen therapy due to SARS-CoV-2 or patient who are on chronic oxygen therapy due to an underlying condition not related to SARS-CoV-2 that require an increase in oxygen flow rate from baseline (Taylor et al., 2021). Furthermore, the FDA EUAs indicates that all approved mAbs may be associated with worse clinical outcomes when administered to hospitalized patients with SARS-CoV-2 requiring high flow oxygen or mechanical ventilation. In the bamlanivimab plus etesevimab arm, the trial showed a 4.8% absolute reduction and a 70% relative reduction in hospitalizations due to SARS-CoV-2 or deaths from any cause. The authorized dosage of 700/1400 mg lower than the dosage tested in BLAZE-1 is based on initial results (Dougan et al., 2021). Sotrovimab is supported by the results of an interim analysis of an ongoing multicenter, double-blind, Phase 3 COMETICE trial (NCT04545060) (Gupta et al., 2021). The main limitation of these studies is the reported result of environmental heterogeneity, making it difficult to make appropriate comparisons are shown in **Table 6**.

**Table 6:** Randomized clinical trials supporting mAbs approved by FDA EUAs.

Monoclonal antibody	Clinical trial number	Study Design	Methods		Results		References
			Intervention:	Primary endpoint	Number of Participants	Primary outcome	
Bamlanivimab plus etesevimab	(Trial Number NCT04427501)	Double-blind, phase 3 randomized clinical trial in outpatients with mild to moderate SARS-CoV-2 who are at high risk for progressing to severe SARS-CoV-2 and/or hospitalization	Single intravenous infusion of etesevimab 2800 mg - Placebo with amlanivimab 2800 mg+	Proportion of participants with SARS-CoV-2 related hospitalization or death by any cause by day 29	bamlanivimab + etesevimab (*n* = 518) - placebo (*n* = 517)	Proportion of participants with SARS-CoV-2 related hospitalization in the bamlanivimab + etesevimab	Dougan et al., 2021
Casirivimab plus imdevimab	NCT04425629	Double-blind, Phase 3 RCT in outpatients with mild to moderate SARS-CoV-2	Single intravenous infusion of: - casirivimab 600 mg + imdevimab 600 mg - casirivimab 1200 mg + imdevimab 1200 mg	Proportion of patients with SARS-CoV-2-related hospitalization or all-cause death through Day 29	SARS-CoV-2-related hospitalization or all-cause death through Day 29	Casirivimab 600 mg + imdevimab 600 mg (*n* = 736) ; 7 of 736 (1.0%) in casirivimab 600 mg plus imdevimab 600 mg; 18 of 1355 (1.3%) in casirivima62 of 1341	Bierle et al., 2021
Sotrovimab	NCT04545060	Double-blind, Phase 1/2/3 RCT in outpatients with mild to moderate SARS-CoV-2	Sotrovimab 500 mg IV - Placebo	Proportion of patients with hospitalization or death from any cause by Day 29	Proportion of patients with hospitalization or death from any cause by Day 29	Sotrovimab (*n* = 291) placebo (*n* = 292); There an 85% relative risk reduction in all-cause hospitalizations	Gupta et al., 2021

## Personalized Cell Therapies to Combat SARS-CoV-2

**Figure 3:**
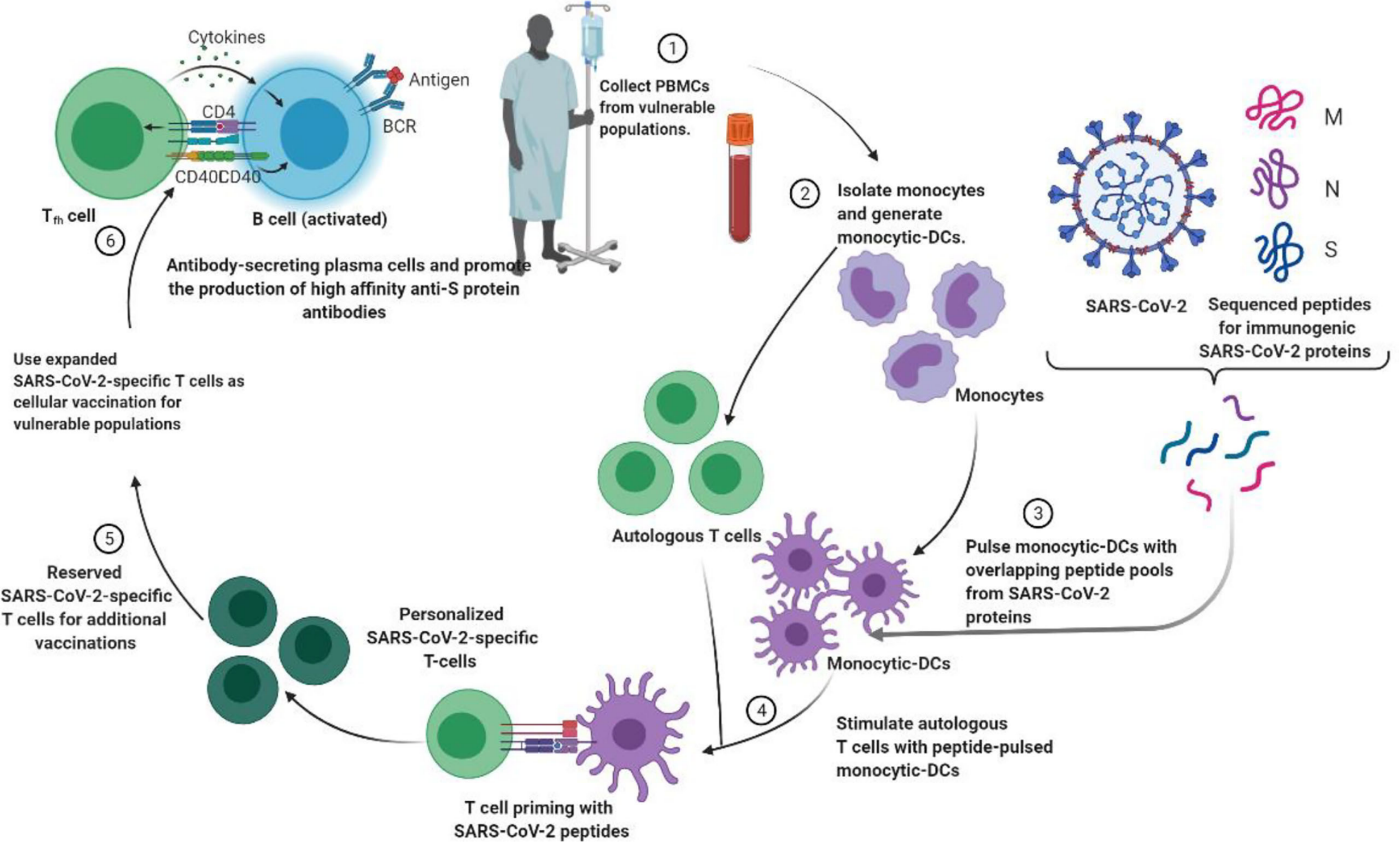
Strategies to generate tailored virus-specific T cells as potential therapeutics for prophylaxis and/or treatment of SARS-CoV-2 infection among vulnerable populations. Monocyte DCs from individuals are treated with SARS-CoV-2 peptide and then used to prime T cells from the same individual to generate SARS-CoV-2-specific T cells. These T cells can be cryopreserved or injected into vulnerable people to prevent or treat SARS-CoV-2.

Personalized medicine plays an important role in the treatment of 19 cases of severe COVID (Khoury et al., 2020; Toor et al., 2021). The idea of cell-based treatment has not been accepted by some scientific communities due to some concerns about the lack of satisfactory clinical research. Nonetheless, MSC and its clinical results show the safety and efficacy of this therapeutic approach in some diseases, especially immune-inflammatory and some incurable diseases (**Figure 3**) (Khoury et al., 2020; Li et al., 2020). With promising results, clinical trials are ongoing. Currently, there are no approved cell-based therapies to prevent or treat patients with SARS-CoV-2 virus, and various clinical studies are underway. Recently, MSCs (Mesenchymal Stem Cells) have attracted clinical trials because of their immunomodulatory properties (Toor et al., 2021). Moreover, as long as the MSC is clinical and time consuming and costly, the MSC remains suspicious.

In mRNA vaccines, single-strand RNA (ssRNA) and double-strand RNA (dsRNA) is recognized by endosomes and cytosols. which is an important part of the natural immune response to the virus (Park et al., 2021). Endosomal Toll-like receptors bind to endosome ssRNA and inflammasome components such as MDA5, RIGI, NOD2 and PKR. Inflammasome components activate the production of interferons and inflammation Mediators. Current vaccines contain purified *in vitro* transcriptional single-stranded mRNA with nucleotides modified to reduce binding to TLRs and immune sensors, thus inhibiting overproduction of type I interferon and cell translation. Functions are limited. LNP carriers further protect mRNA, target delivery to lymphatic vessels, and promote protein translation in lymphatic vessels. Preclinical and early results from human studies show that both vaccines produce anti-S protein IgG and virus-specific neutralizing antibody responses months after vaccination, but T cell data not been completely elucidated (Teijaro and Farber, 2021).

## Prophylactic Use of mAb Against SARS-CoV-2

Vaccines are the most effective way for most people to protect themselves from COVID 19. For the past two years, as the only possible solution to the further spread and recurrence of SARS-CoV-2, the entire scientific community has focused on researching, developing, and ultimately manufacturing safe and effective vaccines (Liz et al., 2020; Nathan et al., 2021). Vaccine development can take years or even decades, but aggressive efforts to screen multiple COVID19 vaccine candidates simultaneously can significantly reduce the overall time required for the development process. MAb is currently an alternative preventive route for COVID19 and may provide short-term prophylaxis to those who have not yet been vaccinated or who do not respond appropriately to vaccination, such as immunocompromised patients. In addition, mAb may be useful if the circulating mutant virus is not adequately covered by vaccination protection (Dong et al., 2021). The PROVENT study was conducted on subjects who would benefit from long-acting antibody prevention because of an increased risk of inadequate response to active immunization or an increased risk of SARS-CoV-2 infection.

## Monitoring Resistance to mAbs Among the New Variants

Monitoring resistance to mAbs among new mutants is important in deciding whether to discontinue some of the newly developed mAbs or investigate different combinations. Mutations in SARS-CoV-2 peplomer and clinical mAb resistance profile in VOC It is summarized in **Table 7**.

**Table 7:** Mutations of SARS-CoV-2 S in VOC and resistance profile of clinical mAbs.

VOC	Bamlanivimab	Etesevimab	Casirivimab	Indevimab	Sotrovimab	Cilgavimab	Tixagevimab	Regdanvimab
B.1.351 (South Africa)	Resistant	R	Resistant	Sensitive	Sensitive	Sensitive	Sensitive	Poorly neutralized or not neutralized
B.1.1.7 (UK)	Sensitive	Sensitive	Sensitive	Sensitive	Sensitive	Sensitive	Sensitive	Sensitive
P.1 (Brazil)	Resistant	Resistant	Resistant	Sensitive	Sensitive	Sensitive	Sensitive	Poorly neutralized or not neutralized
B.1.1.258 (Scotland)	Sensitive	Not known	Sensitive	Resistant	Sensitive	Not known	Not known	Poorly neutralized or not neutralized
B.1.526 (New York)	Potential Sensitive pot	Potential Sensitive pot	Potential Sensitive pot	Potential Sensitive pot	Sensitive	Potential Sensitive pot	Potential Sensitive pot	Not known
B.1.617.1 (India)	Resistant	Sensitive	Sensitive	Sensitive	Sensitive	Potential Sensitive pot	Potential Sensitive pot	Not known
B.1.525 (Nigeria)	Poorly neutralized or not neutralized	Poorly neutralized or not neutralized	Potential Sensitive pot	Potential Sensitive pot	Sensitive	Potential Sensitive pot	Potential Sensitive pot	Not known
B.1.429 (California)	Resistant	Sensitive	Sensitive	Sensitive	Sensitive	Sensitive	Sensitive	Poorly neutralized or not neutralized

## Conclusion

The idea behind the development of SARS-CoV-2 Abs was that enhanced neutralization efficacy would equate to more therapeutic benefit. Development of deactivating cross-reactive human Abs to conserved epitopes on SARS-CoV-2 that can impede infection by emerging SARS-CoV-2 outbreaks. Identification of such conserved epitopes is also essential for the layout of broadly reactive vaccines to thwart future SARS-related coronavirus infections. Monoclonal antibodies and neutralizing antibodies targeting SARS-CoV-2 virus antigens have shown promising results in treating SARS-CoV-2 patients and controlling disease progression. To improve treatment options, an effective understanding of the competent therapeutic characteristics of antibody-based treatments, primarily neutralizing monoclonal antibodies and establishing their therapeutic or prophylactic applications against SARS-CoV-2, is required. In addition, among other potential therapeutic strategies, personalized viral-specific T cells can be generated to prevent infections among populations at risk and/or treat SARS-CoV-2 infections.

## Author Contributions

DDS and DKY conceived and designed the project, collected data from the literature. DDS, AS, H-JL, and DKY analyzed the data and wrote the manuscript. All authors have read and approved the final version of the manuscript.

## Funding

This study is supported by the GRRC program of Gyeonggi province South Korea: GRRC-Gachon 2020(B03), Development of Healthcare Contents based on AI and partly supported by Gachon University Research Fund of 2018(GCU-2018-0369).

## Conflict of Interest

The authors declare that the research was conducted in the absence of any commercial or financial relationships that could be construed as a potential conflict of interest.

## Publisher’s Note

All claims expressed in this article are solely those of the authors and do not necessarily represent those of their affiliated organizations, or those of the publisher, the editors and the reviewers. Any product that may be evaluated in this article, or claim that may be made by its manufacturer, is not guaranteed or endorsed by the publisher.

## References

[B1] Aschenbrenner D. S. (2021). Another Monoclonal Antibody Granted EUA to Treat SARS-COV-2. AJN. Am. J. Nurs. 121 (8), 22. doi: 10.1097/01.NAJ.0000767788.97481.65 34819468

[B2] Ashraf M. U. Kim Y. Kumar S. Seo D. Ashraf M. Bae Y. S. (2021). COVID-19 Vaccines (Revisited) and Oral-Mucosal Vector System as a Potential Vaccine Platform. Vaccines (Basel). 9 (2), 171. doi: 10.3390/vaccines9020171 33670630 PMC7922043

[B3] Baral P. K. Yin J. James M. N. (2021). Treatment and Prevention Strategies for the COVID 19 Pandemic: A Review of Immunotherapeutic Approaches for Neutralizing SARS-CoV-2. Int. J. Biol. Macromol. 186, 490–500. doi: 10.1016/j.ijbiomac.2021.07.013 34237371 PMC8256663

[B4] Bierle D. M. Ganesh R. Razonable R. R. (2021). Breakthrough SARS-COV-2 and Casirivimab-Imdevimab Treatment During a SARS-CoV-2 B. 1.617. 2 (Delta) Surge. J. Clin. Virol. 145, 105026. doi: 10.1016/j.jcv.2021.105026 34775142 PMC8574126

[B5] Bonaventura A. Vecchié A. Wang T. S. Lee E. Cremer P. C. Carey B.. (2020). Targeting GM-CSF in SARS-COV-2 Pneumonia: Rationale and Strategies. Front. Immunol. 11, 1625. doi: 10.3389/fimmu.2020.01625 32719685 PMC7348297

[B6] Callaway E. (2021). Heavily Mutated Coronavirus Variant Puts Scientists on Alert. Nature 600 (7887), 21. doi: 10.1038/d41586-021-03552-w 34824381

[B7] Cheng Q. Chen J. Jia Q. Fang Z. Zhao G. (2021). Efficacy and Safety of Current Medications for Treating Severe and non-Severe SARS-COV-2 Patients: An Updated Network Meta-Analysis of Randomized Placebo-Controlled Trials. Aging (Albany. NY). 13, 21866. doi: 10.18632/aging.203522 34531332 PMC8507270

[B8] Chen N. Zhou M. Dong X. Qu J. Gong F. Han Y.. (2020). Epidemiological and Clinical Characteristics of 99 Cases of 2019 Novel Coronavirus Pneumonia in Wuhan, China: A Descriptive Study. Lancet 395, 507–513. doi: 10.1016/S0140-6736(20)30211-7 32007143 PMC7135076

[B9] Deeks E. D. (2021). Casirivimab/Imdevimab: First Approval. Drugs 81 (17), 2047–2055. doi: 10.1007/s40265-021-01620-z 34716907 PMC8556815

[B10] Della-Torre E. Campochiaro C. Cavalli G. De Luca G. Napolitano A. La Marca S.. (2020). Interleukin-6 Blockade With Sarilumab in Severe SARS-COV-2 Pneumonia With Systemic Hyperinflammation: An Open-Label Cohort Study. Ann. Rheum. Dis. 79, 1277–1285. doi: 10.1136/annrheumdis-2020-218122 32620597 PMC7509526

[B11] Diamond M. Chen R. Winkler E. Case J. Aziati I. Bricker T.. (2021). *In Vivo* Monoclonal Antibody Efficacy Against SARS-CoV-2 Variant Strains. Res. Square. rs.3.rs-448370. doi: 10.21203/rs.3.rs-448370/v1 PMC8349859 34153975

[B12] Doggrell S. A. (2021). Do We Need Bamlanivimab? Is Etesevimab a Key to Treating SARS-CoV-2? Expert Opin. Biol. Ther. 21 (11), 1359–1362. doi: 10.1080/14712598.2021.1985458 34555986 PMC8500303

[B13] Dong J. Zost S. J. Greaney A. J. Starr T. N. Dingens A. S. Chen E. C.. (2021). Genetic and Structural Basis for SARS-CoV-2 Variant Neutralization by a Two-Antibody Cocktail. Nat. Microbiol. 6, 1233–1244. doi: 10.1038/s41564-021-00972-2 34548634 PMC8543371

[B14] Dougan M. Nirula A. Azizad M. Mocherla B. Gottlieb R. L. Chen P.. (2021). Bamlanivimab Plus Etesevimab in Mild or Moderate SARS-CoV-2. New Engl. J. Med. 385, 1382–1392. doi: 10.1056/NEJMoa2102685 34260849 PMC8314785

[B15] Felgenhauer U. Schoen A. Gad H. H. Hartmann R. Schaubmar A. R. Failing K.. (2020). Inhibition of SARS–CoV-2 by Type I and Type III Interferons. J. Biol. Chem. 295, 13958–13964. doi: 10.1074/jbc.AC120.013788 32587093 PMC7549028

[B16] Focosi D. Tuccori M. Baj A. Maggi F. (2021). SARS-CoV-2 Variants: A Synopsis of *In Vitro* Efficacy Data of Convalescent Plasma, Currently Marketed Vaccines, and Monoclonal Antibodies. Multidiscip. Digital. Publish. Inst. 13 (7), 1211. doi: 10.3390/v13071211 PMC8310233 34201767

[B17] Gremese E. Cingolani A. Bosello S. L. Alivernini S. Tolusso B. Perniola S.. (2020). Sarilumab Use in Severe SARS-CoV-2 Pneumonia. EClinicalMedicine 27, 100553. doi: 10.1016/j.eclinm.2020.100553 33043284 PMC7531933

[B18] Gupta A. Gonzalez-Rojas Y. Juarez E. Crespo Casal M. Moya J. Falci D. R.. (2021). Early Treatment for SARS-CoV-2 With SARS-CoV-2 Neutralizing Antibody Sotrovimab. N Engl. J. Med. 385, 1941–1950. doi: 10.1056/NEJMoa2107934 34706189

[B19] Haimei M. (2020). Pathogenesis and Treatment Strategies of SARS-COV-2-Related Hypercoagulant and Thrombotic Complications. Clin. Appl. Thrombosis/Hemostasis. 26, 1076029620944497. doi: 10.1177/1076029620944497 PMC7391437 32722927

[B20] Haljasmägi L. Salumets A. Rumm A. P. Jürgenson M. Krassohhina E. Remm A.. (2020). Longitudinal Proteomic Profiling Reveals Increased Early Inflammation and Sustained Apoptosis Proteins in Severe SARS-COV-2. Sci. Rep. 10, 1–12. doi: 10.1038/s41598-020-77525-w 33239683 PMC7689507

[B21] Hermine O. Mariette X. Tharaux P.-L. Resche-Rigon M. Porcher R. Ravaud P.. (2021). Effect of Tocilizumab vs Usual Care in Adults Hospitalized With SARS-COV-2 and Moderate or Severe Pneumonia: A Randomized Clinical Trial. JAMA Internal Med. 181, 32–40. doi: 10.1001/jamainternmed.2020.6820 33080017 PMC7577198

[B22] Kar S. K. Ransing R. Arafat S. Y. Menon V. (2021). Second Wave of SARS-COV-2 Pandemic in India: Barriers to Effective Governmental Response. EClinicalMedicine 36, 100915. doi: 10.1016/j.eclinm.2021.100915 34095794 PMC8164526

[B23] Khoury M. Cuenca J. Cruz F. F. Figueroa F. E. Rocco P. R. M. Weiss D. J. (2020). Current Status of Cell-Based Therapies for Respiratory Virus Infections: Applicability to SARS-COV-2. Eur. Respir. J. 55, 2000858. doi: 10.1183/13993003.00858-2020 32265310 PMC7144273

[B24] Kim C. Ryu D. K. Lee J. Kim Y. I. Seo J. M.. (2021). A Therapeutic Neutralizing Antibody Targeting Receptor Binding Domain of SARS-CoV-2 Spike Protein. *Nature Communications* 12, 288. doi: 10.1038/s41467-020-20602-5 PMC7803729 33436577

[B25] Koyama T. Platt D. Parida L. (2020). Variant Analysis of SARS-CoV-2 Genomes. Bull. World Health Organ. 98, 495. doi: 10.2471/BLT.20.253591 32742035 PMC7375210

[B26] Kumar S. Sarthi P. Mani I. Ashraf M. U. Kang M. H. Kumar V.. (2021). Epitranscriptomic Approach: To Improve the Efficacy of ICB Therapy by Co-Targeting Intracellular Checkpoint CISH. Cells 10 (9), 2250. doi: 10.3390/cells10092250 34571899 PMC8466810

[B27] Kuritzkes D. R. (2021). Bamlanivimab for Prevention of SARS-COV-2. JAMA 326 (1), 31–32. doi: 10.1001/jama.2021.7515 34081075

[B28] Li Z. Niu S. Guo B. Gao T. Wang L. Wang Y.. (2020). Stem Cell Therapy for SARS-COV-2, ARDS and Pulmonary Fibrosis. Cell Prolif. 53, e12939. doi: 10.1111/cpr.12939 33098357 PMC7645923

[B29] Liu Z. Wu H. Egland K. A. Gilliland T. C. Dunn M. D. Luke T. C.. (2021). Human Immunoglobulin From Transchromosomic Bovines Hyperimmunized With SARS-CoV-2 Spike Antigen Efficiently Neutralizes Viral Variants. Hum. Vaccines Immunotherapeut. 1–10. doi: 10.1080/21645515.2021.1940652 PMC8290372 34228597

[B30] Mahase E. (2021). Covid-19: AstraZeneca Says Its Antibody Drug AZD7442 Is Effective For Preventing and Reducing Severe Illness. BMJ 375, 2860. doi: doi: 10.1136/bmj.n2860 34799345

[B31] Murugan C. Ramamoorthy S. Guruprasad K. Murugan R. K. Sivalingam Y. Sundaramurthy A. (2021). SARS-COV-2: A Review of Newly Formed Viral Clades, Pathophysiology, Therapeutic Strategies and Current Vaccination Tasks. Int. J. Biol. Macromol. 193 (Pt B), 1165–1200. doi: doi: 10.1016/j.ijbiomac.2021.10.144 34710479 PMC8545698

[B32] Nathan R. Shawa I. de la Torre I. Pustizzi J. M. Haustrup N. Patel D. R.. (2021). A Narrative Review of the Clinical Practicalities of Bamlanivimab and Etesevimab Antibody Therapies for SARS-CoV-2. Infect. Dis. Ther. 10, 1933–1947. doi: 10.1007/s40121-021-00515-6 34374951 PMC8353431

[B33] Nayak B. Lal G. Kumar S. Das C. J. Saraya A. (2021). Host Response to SARS-CoV-2and Emerging Variants in Pre-Existing Liver and Gastrointestinal Diseases. Front. Cell. Infect. Microbiol. 11, 753249. doi: 10.3389/fcimb.2021.753249 34760721 PMC8573081

[B34] O'Brien M. P. Forleo-Neto E. Sarkar N. Isa F. Hou P. Chan K. C.. (2021). Subcutaneous REGEN-COV Antibody Combination in Early SARS-CoV-2 Infection. medRxiv. preprint. Server. Health Sci. 2021.06.14.21258569. doi: 10.1101/2021.06.14.21258569

[B35] Park J. W. Lagniton P. Liu Y. Xu R. H. (2021). mRNA Vaccines for SARS-COV-2: What, Why and How. Int. J. Biol. Sci. 17 (6), 1446–1460. doi: 10.7150/ijbs.59233 33907508 PMC8071766

[B36] Perlin D. S. Zafir-Lavie I. Roadcap L. Raines S. Ware C. F. Neil G. A. (2020). Levels of the TNF-Related Cytokine Light Increase in Hospitalized SARS-COV-2 Patients With Cytokine Release Syndrome and ARDS. MSphere 5, e00699–e00620. doi: 10.1128/mSphere.00699-20 PMC7426176 32817460

[B37] Quiros-Roldan E. Amadasi S. Zanella I. Degli Antoni M. Storti S. Tiecco G.. (2021). Monoclonal Antibodies Against SARS-CoV-2: Current Scenario and Future Perspectives. Pharmaceut. (Basel. Switzerland). 14 (12), 1272. doi: 10.3390/ph14121272 PMC8707981 34959672

[B38] Razonable R. R. Pawlowski C. O'horo J. C. Arndt L. L. Arndt R. Bierle D. M.. (2021). Casirivimab–Imdevimab Treatment is Associated With Reduced Rates of Hospitalization Among High-Risk Patients With Mild to Moderate Coronavirus Disease-19. EClinicalMedicine 40, 101102. doi: 10.1016/j.eclinm.2021.101102 34485873 PMC8404031

[B39] Rodriguez-Perez A. I. Labandeira C. M. Pedrosa M. A. Valenzuela R. Suarez-Quintanilla J. A. Cortes-Ayaso M.. (2021). Autoantibodies Against ACE2 and Angiotensin Type-1 Receptors Increase Severity of SARS-COV-2. J. Autoimmun. 122, 102683. doi: 10.1016/j.jaut.2021.102683 34144328 PMC8193025

[B40] Saeed M. Saeed A. Alam M. J. Alreshidi M. (2020). Identification of Persuasive Antiviral Natural Compounds for SARS-COV-2 by Targeting Endoribonuclease NSP15: A Structural-Bioinformatics Approach. Mol. (Basel. Switzerland). 25 (23), 5657. doi: 10.3390/molecules25235657 PMC7729992 33271751

[B41] Samaee H. Mohsenzadegan M. Ala S. Maroufi S. S. Moradimajd P. (2020). Tocilizumab for Treatment Patients With SARS-COV-2: Recommended Medication for Novel Disease. Int. Immunopharmacol. 89, 107018. doi: 10.1016/j.intimp.2020.107018 33045577 PMC7494278

[B42] Sette A. Crotty S. (2021). Adaptive Immunity to SARS-CoV-2 and SARS-COV-2. Cell 184 (4), 861–880. doi: 10.1016/j.cell.2021.01.007 33497610 PMC7803150

[B43] Singh D. D. Han I. Choi E.-H. Yadav D. K. (2020a). Immunopathology, Host-Virus Genome Interactions, and Effective Vaccine Development in SARS-CoV-2. Comput. Struct. Biotechnol. J. 18, 3774–3787. doi: 10.1016/j.csbj.2020.11.011 33235690 PMC7677077

[B44] Singh D. D. Han I. Choi E.-H. Yadav D. K. (2020b). Recent Advances in Pathophysiology, Drug Development and Future Perspectives of SARS-CoV-2. Front. Cell Dev. Biol. 8, 1124. doi: 10.3389/fcell.2020.580202 PMC7677140 33240881

[B45] Singh D. D. Parveen A. Yadav D. K. (2021). SARS-CoV-2: Emergence of New Variants and Effectiveness of Vaccines. Front. Cell. Infect. Microbiol. 11, 777212. doi: 10.3389/fcimb.2021.777212 34970509 PMC8713083

[B46] Stone J. H. Frigault M. J. Serling-Boyd N. J. Fernandes A. D. Harvey L. Foulkes A. S.. (2020). Efficacy of Tocilizumab in Patients Hospitalized With SARS-CoV-2. N Engl. J. Med. 383, 2333–2344. doi: 10.1056/NEJMoa2028836 33085857 PMC7646626

[B47] Syed Y. Y. (2021). Regdanvimab: First Approval. Drugs 81 (18), 2133–2137. doi: 10.1007/s40265-021-01626-7 34724174 PMC8558754

[B48] Taylor P. C. Adams A. C. Hufford M. M. de la Torre I. Winthrop K. Gottlieb R. L. (2021). Neutralizing Monoclonal Antibodies for Treatment of SARS-COV-2. Nat. Rev. Immunol. 21, 382–393. doi: 10.1038/s41577-021-00542-x 33875867 PMC8054133

[B49] Teijaro J. R. Farber D. L. (2021). SARS-COV-2 Vaccines: Modes of Immune Activation and Future Challenges. Nat. Rev. Immunol. 21 (4), 195–197. doi: 10.1038/s41577-021-00526-x 33674759 PMC7934118

[B50] Temesgen Z. Burger C. D. Baker J. Polk C. Libertin C. Kelley C.. (2021a). Lenzilumab Efficacy and Safety In Newly Hospitalized Sars-Cov-2 Subjects: Results from the Live-Air Phase 3 Randomized Double-Blind Placebo-Controlled Trial. medRxiv. doi: 10.1101/2021.05.01.21256470

[B51] Temesgen Z. Kenderian S. S. Badley A. D. (2021b). In Reply — Clinical Benefit of Lenzilumab in Cases of Coronavirus Disease 2019. Mayo Clin Proc 96 (3), 817–818. doi: 10.1016/j.mayocp.2020.12.029 PMC7836950 33673930

[B52] Toor S. M. Saleh R. Sasidharan Nair V. Taha R. Z. Elkord E. (2021). T-Cell Responses and Therapies Against SARS-CoV-2 Infection. Immunology 162, 30–43. doi: 10.1111/imm.13262 32935333 PMC7730020

[B53] Wang C. Li W. Drabek D. Okba N. M. Van Haperen R. Osterhaus A. D.. (2020). A Human Monoclonal Antibody Blocking SARS-CoV-2 Infection. Nat. Commun. 11, 1–6. doi: 10.1038/s41467-020-16256-y 32366817 PMC7198537

[B54] Winkler E. S. Gilchuk P. Yu J. Bailey A. L. Chen R. E. Chong Z.. (2021). Human Neutralizing Antibodies Against SARS-CoV-2 Require Intact Fc Effector Functions for Optimal Therapeutic Protection. Cell 184, 1804–1820. e1816. doi: 10.1016/j.cell.2021.02.026 33691139 PMC7879018

[B55] Yadav D. K. Singh D. D. Han I. Kumar Y. Choi E. H. (2021). Current Potential Therapeutic Approaches Against SARS-CoV-2: A Review. Biomedicines 9 (11), 1620. doi: 10.3390/biomedicines9111620 34829850 PMC8615922

[B56] Yang M. Li J. Huang Z. Li H. Wang Y. Wang X.. (2021). Structural Basis of a Human Neutralizing Antibody Specific to the SARS-CoV-2 Spike Protein Receptor-Binding Domain. Microbiol. Spectr. 9, e01352–e01321. doi: 10.1128/Spectrum.01352-21 PMC8515945 34643438

[B57] Yang L. Liu W. Yu X. Wu M. Reichert J. M. Ho M. (2020). SARS-COV-2 Antibody Therapeutics Tracker: A Global Online Database of Antibody Therapeutics for the Prevention and Treatment of SARS-COV-2. Antibody. Ther. 3, 205–212. doi: 10.1093/abt/tbaa020 PMC7454247 33215063

